# Novel oxazolidinones harbor potent *in vitro* activity against the clinical isolates of multidrug-resistant *Mycobacterium tuberculosis* in China

**DOI:** 10.3389/fmed.2022.1067516

**Published:** 2022-11-29

**Authors:** Chenqian Wang, Guirong Wang, Fengmin Huo, Yi Xue, Junnan Jia, Lingling Dong, Liping Zhao, Fen Wang, Hairong Huang, Hongfei Duan

**Affiliations:** ^1^Department of Tuberculosis, Beijing Chest Hospital, Capital Medical University, Beijing, China; ^2^National Clinical Laboratory on Tuberculosis, Beijing Key Laboratory on Drug-Resistant Tuberculosis, Beijing Chest Hospital, Capital Medical University, Beijing, China

**Keywords:** oxazolidinone, *Mycobacterium tuberculosis*, non-tuberculous mycobacteria, drug resistance, minimum inhibitory concentration

## Abstract

**Objective:**

To investigate the *in vitro* activities of five oxazolidinones in parallel against the reference strains of different mycobacterial species and clinical isolates of *Mycobacterium tuberculosis* (Mtb), and shed light on the differences in the efficacy of these homolog drugs.

**Materials and methods:**

The minimum inhibitory concentrations (MICs) of linezolid, tedizolid, sutezolid, delpazolid, and contezolid against 16 mycobacterial reference strains and 69 *M. tuberculosis* clinical isolates, including 17 drug-susceptible isolates and 52 multidrug-resistant (MDR) isolates, were determined by microplate alamarBlue assay (MABA). The intracellular killing activities of contezolid and linezolid against Mtb H37Rv were compared. In addition, mutations in the linezolid resistance-related genes (*rplC*, *rplD*, and 23S rRNA) of the Mtb clinical isolates were also analyzed.

**Results:**

Tedizolid exhibited the strongest inhibitory activities against the reference strains of both rapidly growing mycobacteria (RGM) and slowly growing mycobacteria (SGM), among the tested oxazolidinones. In contrast, sutezolid only manifested potent activity against reference strains of SGM. Linezolid, delpazolid, and contezolid were less active against the non-tuberculous mycobacterial references. For the Mtb clinical isolates, the antimicrobial action was ranked as: sutezolid > tedizolid > contezolid and linezolid > delpazolid, whereas no difference between drug-sensitive and multiple drug-resistant isolates was observed. Notably, contezolid demonstrated obviously superior intracellular antimicrobial activity than linezolid. Few strains harbored mutations in *rrl* gene or *rplD* genes, although these strains had drug susceptible profiles to linezolid.

**Conclusion:**

Different oxazolidinones can have discrepant antimicrobial activity against different mycobacterial species, or have different manifestations out of cell or in cell. Understanding these differences would be helpful in choosing the appropriate drug in clinical practice.

## Introduction

Tuberculosis (TB) is a chronic infectious disease caused by *Mycobacterium tuberculosis* (Mtb), which remains a huge global public health threat for decades. According to World Health Organization (WHO) Global Tuberculosis Report (1), tuberculosis resulted in ∼1.60 million deaths in 2021. In contrast with the drug-susceptible tuberculosis (DS-TB), multidrug-resistant tuberculosis (MDR-TB), and extensively drug-resistant tuberculosis (XDR-TB) incur longer treatment courses, higher costs, and greater drug side-effect. These factors significantly hamper the global tuberculosis control program. Therefore, novel anti-mycobacterial drugs with great safety, tolerability, and efficacy are crucial in curbing tuberculosis, especially drug-resistant tuberculosis.

Oxazolidinones are a relatively new class of synthetic antibiotics that have shown potent activities against drug-resistant Gram-positive bacteria as well as Mtb. Oxazolidinones inhibit bacterial protein synthesis by competitively binding to the 23S rRNA of the bacterial 50S ribosomal subunit and have no cross-resistance to the existing antibacterial agents. Linezolid (LZD) is the first oxazolidinone drug that is used in TB treatment and has been recommended by WHO as the core drug (group A) for the treatment of drug-resistant TB (2). In addition to its very strong efficacy, adverse reactions (such as bone marrow suppression, peripheral neuropathy, and optic nerve damage) associated with LZD raise great concerns. Therefore, LZD must be reconstructed urgently for ensuring its efficacy and overcoming the associated safety problems. A series of oxazolidinones have been developed in recent years, including tedizolid (TZD), sutezolid (SZD), delpazolid (DZD), contezolid (MRX-I), and others. Among these, TZD is the second oxazolidinone that has been clinically approved for MDR-TB treatment, which manifests stronger activities against both DS-TB and MDR-TB strains than LZD (3, 4). Furthermore, fewer hematological toxicity and neuropathy were observed after long-term TZD treatment in contrast with LZD (5). SZD differs from LZD primarily due to the presence of a thiomorpholine substituent, and better anti-tuberculosis activity and a higher safety profile (6). DZD (LCB01-0371) differs structurally from LZD and contains a cyclic amidrazone that replaces the morpholino ring. Studies have shown that the antibacterial activity of DZD is comparable to LZD in *in vitro* susceptibility tests. In a single ascending dose-based clinical trial (7), DZD at a dose of 2400 mg per day had no serious side effects and exhibited bactericidal and bacteriostatic activity comparable to LZD. In 2021, MRX-I was approved by the Chinese Food and Drug Administration (FDA) to treat Gram-positive bacterial infections. The main active structure of MRX-I is the same like LZD, but it demonstrates less mitochondrial protein synthesis inhibition (MPSi) associated with myelosuppression and less monoamine oxidase inhibition (MAOi) associated with drug-drug interactions. In contrast to LZD (MPSi IC50: 7.9 μg/ml; MAOi IC50: 4.1 μg/ml), MRX-I has lower MPSi (IC50: 15.7 μg/ml), and MAOi (IC50: 12.3 μg/ml). Several studies have shown that MRX-I has comparable *in vitro* and *in vivo* anti-tuberculosis activities to LZD (8). To better understand the potential value of these novel oxazolidinones, we evaluated the *in vitro* antimicrobial inhibitory activity of the above-mentioned four new-generation oxazolidinones and compared them to LZD. The reference strains of different mycobacterial species and clinical isolates of *M. tuberculosis* (Mtb) were included to demonstrate the differences in the efficacy of these homolog drugs. Additionally, the mutations in the reported LZD resistance-related genes (*rplC*, *rplD*, and 23S rRNA) were analyzed to better understand the role of these genes in oxazolidinone resistance. As the second approved oxazolidinone drug for treating drug-resistant TB in China, we paid more attention to MRX-I and determined the intracellular bactericidal effects against Mtb H37Rv in contrast to LZD.

## Materials and methods

### Ethics statement

Because the study involved only laboratory testing with reference strains and clinical isolates, no ethical approval was sought.

### Reference strains and clinical isolates

Totally 69 clinical strains were collected from the strain bank of Beijing Chest Hospital, including 17 DS-TB strains and 52 MDR-TB strains. A total of 16 reference strains of different mycobacterial species were also recruited. All of them originated from the American Type Culture Collection (ATCC).

### Minimum inhibitory concentration testing

The microplate alamarBlue assay (MABA) was performed according to the guidelines of the Clinical and Laboratory Standards Institute (CLSI) to determine the minimum inhibitory concentrations (MICs). The broth microdilution format was set up as a twofold dilution, while the concentrations of all of the tested drugs ranged from 0.0625 to 64 μg/ml. Briefly, fresh culture suspension of 1 McFarland standard was prepared and further diluted (at 1:20) with Middlebrook 7H9 broth containing 10% Middlebrook OADC Enrichment. A total of 100 μl per well of this dilution was added on the 96-well plate. After 7 days of incubation at 37°C, 70 μl of alamarBlue solution was added to each well. Plates were further incubated for 24 h, color changes were then monitored by visual inspection. A change from blue to pink or purple indicated bacterial growth. The MIC was defined as the lowest concentration of antibiotic which prevented a color change from blue to pink. The reference strain Mtb H37Rv was used as a control. The MIC breakpoint concentration was defined as 1.0 μg/ml for LZD, according to the CLSI. All tested oxazolidinones (LZD, MRX-I, DZD, TZD, SZD) came from TargetMOl Chemicals Inc.

### Whole genome sequencing

The genomic DNA of the clinical isolates was extracted by using the MasterPure Complete DNA isolation kit (Epicentre, Madison, WI, USA). DNA libraries were constructed and processed using Illumina kit following the manufacturer’s instructions. Whole genome sequencing (WGS) was performed on an Illumina HiSeq X-Ten sequencing platform. The sequences of genes known to confer resistance to LZD in *M. tuberculosis* (including 23S rRNA, *rplC* and *rplD* genes) were analyzed specifically.

### Intracellular antibacterial assays

The assay was performed according to the method reported previously (9). Bacteria were adjusted to 5 × 10^6^ bacteria per milliliter with RPMI-1640 medium. The monolayers THP-1 cells were infected at a multiplicity of infection of 1:5 (cells:bacilli) and incubated for 4 h at 37°C in a humid atmosphere with 5% CO_2_. In order to remove the extracellular bacteria, cultures were washed twice with pre-warmed PBS at 37°C and then 1 ml of gentamicin (20 μg/ml) was added to each well followed by incubation for 2 h. The wells were washed twice with PBS and 1 ml of RPMI-1640 containing the drug in solution to be tested was added, and the plate was incubated for 24, 48, and 72 h. At the end of the incubation, bacterial counts were determined from each well by agar plating. 7H10 plates were incubated at 37°C for 3–4 weeks until colonies were visible. Three replicates were performed for each concentration of the drugs and bacterial counts of each replicate were done in triplicate.

### Data analysis

MIC_50_, MIC_90_ and epidemiological cutoff (ECOFF) values were calculated using ecofinder-xl-2010-v21-webversion software (CLSI). Data were analyzed using SPSS 26.0 software and GraphPad Prism 8.0 software.

## Results

### MICs of the five oxazolidinones against mycobacterial reference strains

The MICs of the oxazolidinones against the 16 mycobacterial reference strains are shown in Table 1. TZD generally presented the best antimicrobial activities against the enrolled strains, 7 out of 8 rapidly growing mycobacteria (RGM) reference strains and 6 out of 8 slowly growing mycobacteria (SGM) strains had MIC ≤ 0.5 μg/ml. In contrast, SZD only demonstrated likely activity as TZD against SGM but not RGM. LZD, MRX-I, and DZD were probably less active against either RGM or SGM. Oxazolidinones had weak antibacterial activities against *M. avium* and *M. intracellulare* reference strains. Only TZD had relatively strong activity against *M. avium* reference strain whereas SZD had against *M. intracellulare* reference strain. These had MICs of 0.25 and 0.5 μg/ml, respectively. The activity against *M. abscessus* was generic, only TZD had a MIC of 0.5 μg/ml.

**TABLE 1:** MICs of the five oxazolidinones against mycobacterial reference strains.

Reference strain		MIC (μg/ml) of				
		
		LZD	MRX-I	SZD	DZD	TZD
RGM	*M. abscessus subsp.abscessus*	4	16	4	2	0.5
	*M. pulveris*	0.5	1	0.5	2	0.5
	*M. vaccae*	1	1	1	1	0.25
	*M. phlei*	2	2	2	4	1
	*M. smegmatis*	1	2	4	2	0.5
	*M. senegalense*	2	2	1	4	0.5
	*M. cosmeticum*	2	2	8	1	0.5
	*M. flavescens*	2	2	1	4	0.5
SGM	*M. tuberculosis* H37Rv	0.5	1	0.25	2	0.25
	*M. bovis*	0.5	1	0.25	2	0.5
	*M. kansasii*	0.5	1	≤ 0.0625	1	0.125
	*M. africanum*	0.5	1	≤ 0.0625	2	0.125
	*M. avium subsp.avium*	8	8	8	1	0.25
	*M. intracellulare*	16	32	0.5	32	4
	*M. asiaticum*	4	8	0.5	4	1
	*M. parascrofulaceum*	1	2	4	1	0.125

CLSI resistance breakpoint for LZD: *M. tuberculosis* 1 μg/ml; *M. avium* complex, *M. kansasii*, RGM 32 μg/ml.

### MIC distributions of *Mycobacterium tuberculosis* to the five oxazolidinones

**FIGURE 1:**
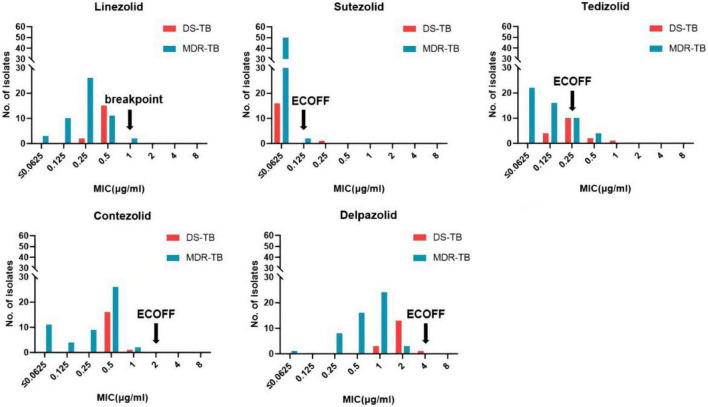
MIC distributions of the five oxazolidinones against 69 Mtb clinical isolates.

The MIC distributions of the five oxazolidinones against the 69 clinical strains of Mtb are shown in Table 2 and Figure 1. The MIC of SZD was 4 to 8 times lower than LZD, whereas the MIC of TZD was 2–4 times lower than LZD. The MIC distribution of MRX-I was similar to LZD, while DZD showed 2–4 times higher MIC than LZD. SZD showed the strongest inhibitory activity among the tested oxazolidinones, with MIC_50_ and MIC_90_ of 0.0625 μg/ml and 0.125 μg/ml, respectively. Notably, the DS- and MDR-TB strains manifested similar MIC distribution profiles. SZD also presented the lowest ECOFFs at 0.125 μg/ml among the five oxazolidinones (Table 2) followed by TZD at 0.25 μg/ml. Notably, the ECOFF for LZD was 1 μg/ml, which is consistent with the CLSI-specified cutoff point for determining LZD resistance. According to the CLSI resistance breakpoint point for LZD, 69 Mtb clinical isolates were sensitive to LZD. For a given isolate, the MICs to different oxazolidinones were correlated (Table 3). With increase of MIC of LZD, the MICs of MRX-I, TZD, and DZD also showed a noticeable upward trend. Such a trend was not observed in the case of SZD.

**TABLE 2:** MICs of the 5 oxazolidinones against 69 Mtb clinical isolates.

Agent	MIC_50_ (μ g/ml)	MIC_90_ (μg/ml)	ECOFF (μg/ml)
Linezolid	0.5	1	1
Contezolid	1	2	2
Sutezolid	0.0625	0.125	0.125
Tedizolid	0.125	0.25	0.25
Delpazolid	2	4	4

**TABLE 3:** MICs of the four novel oxazolidinones for the Mtb clinical isolates with different MICs of linezolid.

Linezolid		No. of isolates with different MICs (μg/ml)			
	
MIC (μg/ml)	No. of isolates	Contezolid	Tedizolid	Sutezolid	Delpazolid
≤ 0.0625	3	≤ 0.0625 (2) 0.25 (1)	≤ 0.0625 (3)	≤ 0.0625 (3)	≤ 0.0625 (1) 0.25 (1) 1 (1)
0.125	10	≤ 0.0625 (3) 0.125 (2) 0.25 (4) 0.5 (1)	≤ 0.0625 (8) 0.125 (1) 0.5 (1)	≤ 0.0625 (10)	0.25 (5) 0.5 (4) 1 (1)
0.25	28	≤ 0.0625 (6) 0.125 (1) 0.25 (4) 0.5 (17)	≤ 0.0625 (11) 0.125 (6) 0.25 (8) 0.5 (2) 1 (1)	≤ 0.0625 (26) 0.125 (2)	0.25 (2) 0.5 (8) 1 (16) 2 (2)
0.5	26	0.125 (1) 0.5 (22) 1 (3)	0.125 (11) 0.25 (12) 0.5 (3)	≤ 0.0625 (25) 0.25 (1)	0.5 (4) 1 (7) 2 (14) 4 (1)
1	2	0.5 (2)	0.125 (2)	≤ 0.0625 (2)	1 (2)

### Mutations conferring linezolid resistance

Although the 69 clinical isolates of Mtb were all susceptible to LZD, WGS identified substitution mutations in the *rrl* gene (C1275T, C2060T, and C2572T) in three strains, whereas 3 other strains harbored a non-synonymous mutation Arg79His (CGT → CAT) in the *rplD* gene. Mutations and corresponding MICs of the five oxazolidinones are shown in Table 4.

**TABLE 4:** Mutations located in *rrl* and *rplD* in six Mtb clinical strains and MICs of five oxazolidinones.

ID	*rrl*	*rplD*	MIC (μ g/ml)				
			
			LZD	MRX-I	SZD	TZD	DZD
15104	C2060T	WT	0.5	0.5	≤ 0.0625	0.25	2
6102	C2572T	WT	0.5	0.5	≤ 0.0625	0.125	2
22222	C1275T	WT	≤ 0.0625	≤ 0.0625	≤ 0.0625	≤ 0.0625	1
13204	WT	G236A (Arg79His)	0.5	0.5	0.25	0.25	2
11263	WT	G236A (Arg79His)	0.25	0.5	≤ 0.0625	≤ 0.0625	1
10181	WT	G236A (Arg79His)	0.25	≤ 0.0625	≤ 0.0625	≤ 0.0625	1

### Intracellular killing activity of contezolid and linezolid against *Mycobacterium tuberculosis* H37Rv

**FIGURE 2:**
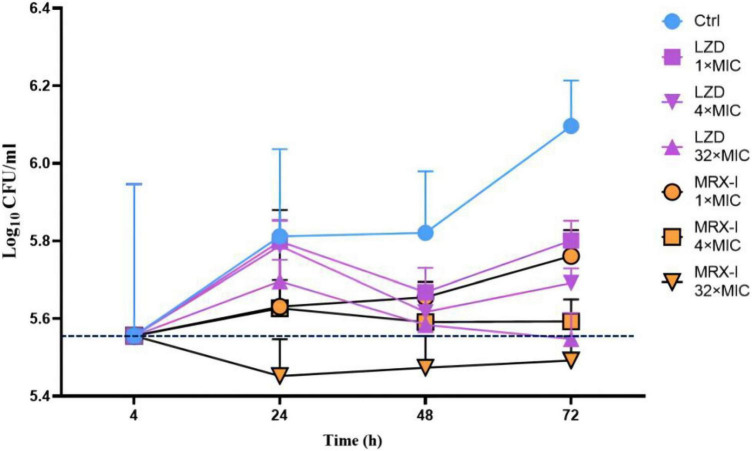
Intracellular activities of antibiotics against H37Rv in the THP-1 macrophage model. Ctrl, control; LZD, Linezolid; MRX-I, Contezolid.

Four hours after H37Rv infection of macrophages, 10% of the bacteria were phagocytosed by macrophages (500,000/5 million) (Figure 2). However, gradually, the intracellular H37Rv also continued to proliferate and proliferated by nearly half after 24 h and more than doubled after 72 h. Both MRX-I and LZD inhibited the intracellular growth of Mtb in contrast with the controls in a dose-dependent manner. MRX-I presented obviously superior intracellular antibacterial effects than LZD. The colony forming units (CFU) counts of MRX-I were lower than LZD at the same drug concentration at each incubation time point. Additionally, only 32 × MIC MRX-I showed bactericidal effects at 24 and 48 h. Furthermore, at 24 h, 1 × MIC MRX-I showed obvious antibacterial activity (*P* < 0.05) while 1 × MIC LZD did not show any antibacterial activity. Even at 4 × MIC, LZD did not demonstrate any obvious bacteriostatic activity.

## Discussion

In the present study, we first compared the MICs of five oxazolidinones side by side against the reference strains of mycobacterial species and Mtb clinical isolates. New generation oxazolidinones have intrigued many interests because of their high efficacy but the toxic features (e.g., of LZD) encountered in clinical usage are a major limitation. DZD had excellent pharmacokinetic parameters and a good safety profile (10). In a recent clinical trial, Choi and colleagues demonstrated that LCB01-0371 (DZD) was well tolerated in healthy male subjects after multiple doses of up to 1,200 mg twice daily for 21 days (11). Several studies have shown that SZD was more potent than LZD in both *in vitro* and *in vivo* assays (12, 13). In addition, it was also much safer than LZD (14–16) and exhibited superior activity against latent tuberculosis (17). Another study has shown that SZD could shorten the treatment course (18). A 14-day preliminary phase II clinical trial (16) demonstrated that SZD at a daily dose of 1200 mg was safe and well tolerated, with high early bactericidal activity. MRX-I was better tolerated and much safer in healthy Chinese subjects in contrast to LZD (19–21). In a 28-day trial, no serious adverse events were observed at 800 or 1,200 mg every 12 h, and none of the patients discontinued the treatment due to any adverse events. MRX-I had a lower incidence of myelosuppression than LZD and did not affect the QT interval at the tested therapeutic dose (800 mg/day) (22, 23). Similarly, in a safety and tolerability study, long-term TZD treatment was associated with lesser hematologic toxicity and neuropathy than LZD (5). From the 81 patients treated with TZD (200 mg once daily) for a median duration of 26.5 days, only 6 patients (7.4%) developed myelosuppression-related thrombocytopenia, whereas none developed peripheral or optic neuropathy or allergic reaction.

In this study, TZD exhibited strong inhibitory activities against the RGM and SGM reference strains, while SZD only manifested potent activity against SGM reference strains. However, inconsistent activities were observed against some of the most frequently isolated non-tuberculous mycobacteria (NTM) species. TZD was the only tested drug in this study that had strong activity against *M. avium* reference strain, while only SZD demonstrated strong activity against *M. intracellulare* reference strain, and only TZD demonstrated strong activity against *M. abscessus* reference strain. Similar findings have also been reported by another study (24). The treatment of NTM infection is always challenging due to the shortage of efficacious drugs. Therefore, whether any of the oxazolidinones could be applied for treating a specific NTM infection is worthy of further investigation.

In accordance with the reference strains, SZD also presented the strongest antibacterial effects on clinical isolates of Mtb, followed by TZD. Both drugs harbored better activities than LZD, while MRX-I had comparable activity to LZD. A previous study (8) showed that the anti-TB effects of SZD and MRX-I were comparable to LZD (with MIC_50_ = 0.5 μg/ml), TZD (MIC_50_ = 0.125 μg/ml) had better anti-tuberculosis effects than LZD. In this assay, DZD (MIC_50_ = 2 μg/ml, MIC_90_ = 4 μg/ml) had the weakest antibacterial effect on Mtb and its MIC was generally 4 times than LZD. Zong Z. et al. (25) showed that the MIC of DZD (MIC_50_ = 0.5 μg/ml) against MDR-TB isolates was about 8 times than LZD (MIC_50_ = 0.064 μg/ml), whereas the MIC values for both drugs were much lower than the MIC values obtained in this study. The main reason for the above differences may due to the difference between the recruited isolates and the operation process of the MIC test.

According to the CLSI critical concentration of LZD (i.e., 1 μg/ml), the 69 clinical isolates of Mtb recruited in this study were all categorized as LZD-sensitive strains. However, we detected some nucleotide substitutions in *rrl* and *rplD* in a few susceptible strains. Since no LZD-resistant isolate was included in this study, we, therefore, conclude that mutations in these genes are plausibly not always related to LZD resistance in *M. tuberculosis*.

Mycobacterial bacilli preferentially reside in cells. A critical step in anti-tuberculosis drug research is the assessment of its intracellular activity against Mtb. A previous study (9) showed that TZD had good intracellular antibacterial activity, with a 1.3 log_10_ CFU/ml reduction in the number of intracellular mycobacteria after 72 h exposure to a drug concentration of 16 μg/ml. In another study (26), SZD was bactericidal against Mtb in macrophages. At the drug concentration of 1, 2, and 4 μg/ml, the intracellular survival number of Mtb was reduced by 2 log over the 8th day of action. In this study, MRX-I exhibited obviously stronger intracellular activity than LZD. Even at 1 × MIC after 24 h of incubation, MRX-I showed obvious antibacterial activity, whereas LZD presented a similar effect only in the test with 32 × MIC after 48 h of incubation. Furthermore, MRX-I presented rapid bactericidal activity. At 32 × MIC after 24 h of incubation, about 0.1 log bacilli number reduction was observed compared with the initial infected bacilli number. However, the bacilli number for LZD at 32 × MIC (after 48 h incubation) was equivalent to the initial invading bacilli number, which indicated that LZD had no bactericidal activity against Mtb. Based on this outcome, and also considering the reported better safety profile, MRX-I becomes ideal for treating drug-resistant TB.

There are some limitations in this study. Firstly, the number of Mtb strains in this experiment was small, and the composition ratio of DS-TB and MDR-TB was not in line with the actual situation, which may affect the reliability of ECOFFs defined in this study. Secondly, due to absence of any LZD-resistant isolate among the enrolled cases, the relationship between mutations in the known drug-resistant genes and oxazolidinone resistance could not be evaluated objectively. Thirdly, due to the very low MIC value of SZD against Mtb, the exact MIC distribution of SZD was not elucidated sufficiently. Therefore, whether or not the MIC values of SZD and other oxazolidinones also have consistent trend remains to be studied in the future.

In conclusion, the novel oxazolidinones exhibited potent antibacterial activity against the reference strains of different species and Mtb clinical isolates, including MDR-TB *in vitro*. As the secondly approved oxazolidinone for drug-resistant treatment in China, contezolid manifested much stronger intracellularly bactericidal activity than linezolid against the Mtb bacilli, which encourages its usage in treating drug-resistant tuberculosis.

## Data availability statement

The original contributions presented in the study are publicly available. This data can be found in the ENA repository, accession number SRP134826.

## Author contributions

CW, GW, and HD participated in the design of the study. CW carried out the experimental studies. CW and HH wrote the manuscript. FH quantitated the drugs. YX, JJ, and LD were responsible for the culture of mycobacteria and THP-1 cells. LZ and FW participated in data analysis. All authors read and approved the final manuscript.
